# Hierarchical Intrafibrillarly Mineralized Collagen Membrane Promotes Guided Bone Regeneration and Regulates M2 Macrophage Polarization

**DOI:** 10.3389/fbioe.2021.781268

**Published:** 2022-01-26

**Authors:** Yaowei Xuan, Lin Li, Muzhi Ma, Junkai Cao, Zhen Zhang

**Affiliations:** ^1^ Medical School of Chinese PLA, Beijing, China; ^2^ Department of Stomatology, The First Medical Centre, Chinese PLA General Hospital, Beijing, China; ^3^ Department of Oral and Maxillofacial-Head and Neck Oncology, Shanghai Ninth People’s Hospital, Shanghai Jiao Tong University School of Medicine, College of Stomatology, Shanghai Jiao Tong University, National Center for Stomatology, National Clinical Research Center for Oral Diseases, Shanghai Key Laboratory of Stomatology, Shanghai, China

**Keywords:** mineralized collagen membrane, osteoimmunomodulation, macrophage polarization, guided bone regeneration, nanostructure

## Abstract

Mineralized collagen has been introduced as a promising barrier membrane material for guided bone regeneration (GBR) due to its biomimetic nanostructure. Immune interaction between materials and host significantly influences the outcome of GBR. However, current barrier membranes are insufficient for clinical application due to limited mechanical or osteoimmunomodulatory properties. In this study, we fabricated hierarchical intrafibrillarly mineralized collagen (HIMC) membrane, comparing with collagen (COL) and extrafibrillarly mineralized collagen (EMC) membranes, HIMC membrane exhibited preferable physicochemical properties by mimicking the nanostructure of natural bone. Bone marrow mesenchymal stem cells (BMSCs) seeded on HIMC membrane showed superior proliferation, adhesion, and osteogenic differentiation capacity. HIMC membrane induced CD206+Arg-1+ M2 macrophage polarization, which in turn promoted more BMSCs migration. In rat skull defects, HIMC membrane promoted the regeneration of new bone with more bone mass and more mature bone architecture. The expression levels of Runx2 and osterix and CD68 + CD206 + M2 macrophage polarization were significantly enhanced. HIMC membrane provides an appropriate osteoimmune microenvironment to promote GBR and represents a promising material for further clinical application.

## Introduction

Regeneration of periodontal tissue is a challenging step in the treatment of periodontitis, especially the regeneration of lost bone tissue. Guided bone regeneration (GBR) refers to the application of barrier membranes to block the ingrowth of gingival epithelium and connective tissue while also inducing the deposition of extracellular matrix, to maximize the repair and regeneration at the periodontal bone tissue (Aprile et al., 2020). The ideal barrier membranes for GBR should possess appropriate characteristics, such as low toxicity, superior biocompatibility, initial mechanical properties, suitable degradation rate, and surface characteristics conducive to cell attachment (Liu et al., 2016). However, among existing barrier materials for GBR, most absorbable membranes lack mechanical properties to maintain the space long enough for bone regeneration, whereas non-absorbable membranes do not degrade and require a second operation for their removal. Thus, both types of membranes are insufficient for clinical application (Zhou et al., 2021).

The fabrication of mineralized collagen materials using biomimetic technology represents a promising approach for GBR due to mimicking the microstructural organization of natural bone. However, hydroxyapatite (HA) crystallites randomly stacked around the collagen fibrils in previously fabricated extrafibrillarly mineralized collagen (EMC), failing to provide an ordered microstructure. Therefore, EMC can offer only analogous chemical composition to native bone but not the same surface appearance and nanostructure of bone extracellular matrix (Kane and Ma, 2013; Hu et al., 2016). In contrast, hierarchical intrafibrillarly mineralized collagen (HIMC), which presents a surface that is highly similar to the natural bone matrix based on a hierarchical combination of collagen and HA, shows superior option for GBR applications (Liu et al., 2016). Both cell and animal studies have verified the excellent osteogenic induction potential and bone regeneration capacity of HIMC material (Liu et al., 2014; Sun et al., 2016; Zhang et al., 2019).

However, most previous studies of biomaterials for bone regeneration emphasized the physical properties and their direct influence on osteocytes, or they focused on materials that induced no response to the immune system to achieve “immune safety”. Through advances in the understanding of osteobiology, immune response was found to be generally activated during interaction between biomaterials and the host. Accordingly, the local microenvironment, especially the immune microenvironment, plays a key role in the regulation of osteogenesis (Schmidt-Bleek et al., 2014; Lin et al., 2021). Macrophages are important components of the immune response to biomaterials and are characterized by high plasticity. M1 macrophages are activated during classic inflammatory response and stimulate the secretion of pro-inflammatory cytokines like interleukin (IL)-6 and tumor necrosis factor alpha (TNF-α) to enhance osteoclast activity and bone resorption. Conversely, M2 macrophages exhibit anti-inflammatory properties and promote bone formation via the production of bone morphogenetic protein-2 and other osteogenic markers (Freytes et al., 2013; Wynn and Vannella, 2016). The ratio and transition of M1/M2 macrophages are considered important indicators of the local immune environment (Yu et al., 2016). Different types of biomaterials, such as collagen (COL) membrane, hydrogel, and biological coating have been shown to regulate macrophage polarization and thereby influence bone regeneration (Chu et al., 2017; Zhang R. et al., 2018; Tanaka et al., 2019). However, the effects of HIMC membrane on immune environment and macrophages comparing with conventional COL and EMC membrane remain relatively undetermined (Shi et al., 2018).

In this study, we investigated whether HIMC membrane can regulate macrophage polarization based on the biomimetic nanostructure. The novelty of the present study lies in the investigation of the osteogenic induction capacity and osteoimmunomodulatory properties elicited by HIMC membrane used for GBR both *in vitro* and *in vivo*. First, HIMC membrane was fabricated, and the physicochemical properties were investigated in comparison to COL and EMC membranes. Moreover, we further explored the osteogenic effectiveness and osteoimmunomodulatory capacity of HIMC membrane using bone marrow-derived mesenchymal stem cells (BMSCs) and rat critical-sized skull defect models, to provide an important experimental basis for further testing the clinical potential of HIMC membrane for GBR.

## Materials and Methods

### Fabrication of Membranes

According to the method of Cui et al. (Sun et al., 2016), hierarchical self-assembled nano-hydroxyapatite (nHA) was guided to nucleate among collagen molecules via a biomimetic mineralization process, resulting in the formation of HIMC membranes (Liao et al., 2004; Lian et al., 2013; Xu et al., 2016). Briefly, nHA was chemically synthesized from calcium salt, sodium hydroxide, and phosphoric acid. By regulating the mineralization process, collagen and HA can be hierarchically self-assembled. EMC membranes were synthesized using a previously described conventional crystallization method (Hu et al., 2016). COL membranes were prepared by dissolving type I collagen in dilute hydrochloric acid and, after adjustment of the pH, applying centrifugal vacuum drying. For *in vitro* experiments, the membranes were manufactured as round samples with diameters of 12 and 30 mm. For *in vivo* study, square samples with side lengths of 9 mm were fabricated.

### Characterization of Membranes

#### Scanning Electron Microscopy

The surface topographies of HIMC, COL, and EMC membranes were surveyed by scanning electron microscopy (Inspect F, FEI, Eindhoven, Netherlands). The samples were fixed with 2.5% glutaraldehyde (pH 7.4) at 4°C for 24 h, rinsed three times with phosphate-buffered saline (PBS), dehydrated in gradient ethanol solutions (30–100%), and dried at critical-point. Then, the samples were gold sputter-coated and viewed under SEM.

#### Fourier Transform Infrared Spectroscopy

We employed FTIR (Thermo Nicole, United States) to investigate the molecular structure and composition of the prepared HIMC, COL, and EMC membranes by analyzing the characteristic absorption peaks of functional groups.

#### Measurement of Mechanical Properties

Five samples of each material were prepared with dimensions of 10 mm × 15 mm × 0.15 mm for tensile strength measurements, and the effective tensile length was 10 mm. We utilized a universal mechanical testing machine (3367; Instron, Norwood, MA) to acquire the stress–strain curves and tensile strength results, with the crosshead speed at 1 mm/min.

#### Water Contact Angle Measurements

The static contact angle between the surface of each membrane type and a water drop was determined using an optical instrument (*n* = 3). Four different droplet points were measured on images taken with a CCD camera to assess the hydrophilicity of each membrane type.

### *In Vitro* Study

#### Cell Culture

The isolation, culture, and identification of rat BMSCs followed the previous study (Zhang Z. et al., 2018). We purchased RAW 264.7 cells from the Cell Resource Center (IBMS, CAMS/PUMC), and the cells were cultured in Dulbecco’s modified Eagle medium (DMEM, Hyclone) containing 10% fetal bovine serum (FBS) in the environment of 37°C humidified incubator with 5% CO_2_.

#### Cell Proliferation Assay

The proliferation of BMSCs seeded on different membranes was estimated using the CCK-8 assay. The membranes were sterilized by ultraviolet light overnight prior to cell seeding. BMSCs (5 × 10^4^ per well) were added over the membranes in 24-well plates (BD Biosciences, Franklin Lakes, NJ, United States). After culture for 1, 3, and 5 days, 50 µl CCK-8 solution (Sigma-Aldrich, United States) was added to each well and incubated for 4 h. Then, the absorbance at 450 nm in each well was detected using a microplate reader (Rayto RT-6000, United States).

#### Cell Adhesion and Morphology

The adhesion and morphology of BMSCs on different membranes were evaluated by SEM and laser scanning confocal microscopy (LSCM) after culture for 24 h. The SEM procedure was the same as described above. For LSCM imaging, BMSCs on the membranes were immunostained to reveal the F-actin cytoskeleton and nucleus. We applied 4% paraformaldehyde to fix the cells, then 0.25% Triton X-100 was applied to permeabilize them, followed by blocking with 1% bovine serum albumin. Anti-F-actin antibody for cytoskeletal protein staining (green) and DAPI for nuclear staining (blue) were used. Representative LSCM images were taken (Olympus, Japan).

#### BMSCs Osteogenic Differentiation

##### Alkaline Phosphatase Assay

ALP assay of BMSCs on membranes was detected after osteogenic culture for 14 days. After removing the medium and rinsing three times with PBS, regents were added following the instructions of the ALP assay kit (Sigma-Aldrich, United States). Finally, ALP staining was conducted and photographed. ALP activity was detected by transferring 50 µl of each sample to a 96-well plate, and the absorbance at 520 nm was measured with an automatic microplate reader.

##### Alizarin Red Staining

Calcium depositions of BMSCs on membranes for 21 days were evaluated by ARS. The samples were fixed in 4% paraformaldehyde after removal of the medium. Deionized water was used to rinse the samples three times and then the staining was performed with 2% ARS solution at room temperature for 20 min. The samples were washed again several times before calcium salt depositions were observed and photographed under an optical microscope. Dye release was quantified with a spectrophotometer at 562 nm.

##### Quantitative Real-Time PCR

We conducted qRT-PCR to evaluate the mRNA expression levels of osteogenic differentiation markers in BMSCs grown on the membranes after 3 days. Total RNA extraction, cDNA synthesis, and qRT-PCR procedure were performed as previously reported (Zhang Z. et al., 2018). Relative mRNA expression levels were calculated by the 2^−ΔΔCt^ method. The primers used for qRT-PCR are presented in Table 1.

**TABLE 1:** Sequences of primers used for qRT-PCR.

Gene	Forward primer sequence (5′–3′)	Reverse primer sequence (5′–3′)
GAPDH	TGT​ATC​TGT​TGT​GGA​TCT​GA	TTG​CTG​TTG​AAG​TCG​CAG​GAG
COLI	AGAACAGCGTAGCCT	TCCGGTGTGACTCGT
OCN	GGA​CCC​TCT​CTC​TGC​TCA​CTC​TG	ACC​TTA​CTG​CCC​TCC​TGC​TTG​G
OPN	TGG​CAG​TGG​TTT​GCT​TTT​GC	TGT​GGT​CAT​GGC​TTT​CAT​TG
CD86	TAA​GCA​AGG​ATA​CCC​GAA​AC	AGA​ATA​CAC​ACA​ATG​GTC​ATA​TT
CD206	AGA​CGA​AAT​CCC​GGC​TAC​GG	CAC​CCA​TTC​GAA​GGC​ACT​C
Arg-1	CTC​CAA​GCC​AAA​GCC​CAT​AGA​G	AGG​GGC​TGT​CAT​TGG​GGA​CAT​C
iNOS	AGA​CCC​AGT​GCC​CTG​CTT​T	CAC​CAA​GGT​CAT​GCG​GCC​T

##### Western Blot

Total proteins were obtained from BMSCs after 3 days in culture using radioimmunoprecipitation assay (RIPA) lysis buffer (Beyotime, China). Then 30 μg protein lysate samples were separated by 8–15% sodium dodecyl sulfate-polyacrylamide gel electrophoresis (SDS-PAGE) before transfer to polyvinylidene difluoride (PVDF) membranes. Then the PVDF membranes were incubated overnight at 4°C with primary antibodies including COLI (AF7001, dilution: 1:200, Affinity Biosciences, United States), OCN (DF12303, dilution: 1:100, Affinity Biosciences, United States), OPN (ab63856, dilution: 1:1,000, Abcam, United States), and GAPDH (ab9484, dilution: 1:200, Abcam, United States). Horseradish peroxidase-conjugated goat anti-rabbit secondary antibody (S0001, dilution: 1:2,000, Affinity Biosciences, United States) was added for incubation for 1 h at room temperature. Finally, enhanced chemiluminescence reagents (Millipore, United States) were utilized to visualize the immunocomplexes.

#### Macrophage Polarization Status

##### Flow Cytometry

RAW 264.7 cells (1 × 10^6^ per well) were seeded on 6-well plates on different membranes. After 24 h, the cells were collected and resuspended in 100 µl binding buffer. M1 macrophage marker CD86 and the M2 marker CD206 antibodies (Biolegend, San Diego, CA, United States) were incubated for 30 min, flow cytometry (Beckman Coulter, Brea, CA, United States) and FlowJo software (TreeStar, United States) were utilized to analyze the cell clusters.

##### qRT-PCR

Total RNA was extracted from RAW 264.7 cells cultured for 24 h. The mRNA expression levels of the *CD86, iNOS, CD206,* and *Arg-1* were analyzed by qRT-PCR. The primers used for qRT-PCR are shown in Table 1.

##### Transwell Migration Assay

The effect of macrophage polarization in response to different membranes on BMSCs migration was investigated by Transwell migration assay. First, conditioned medium was prepared by immersing 500 mg of each material in 50 ml α-MEM for 48 h. In the upper Transwell chamber, BMSCs were seeded, and RAW 264.7 cells were cultured in the lower. After overnight, the medium in the lower chamber was replaced with the material-conditioned medium. After 24 h of incubation, 4% paraformaldehyde was used for 30 min to fix the cells in the upper chamber. Then, the cells were stained with a crystal violet solution for observation and counting of the migrated BMSCs under an optical microscope.

### *In Vivo* Study

#### Rat Critical-Sized Skull Defect Model and GBR Process

Eight-week-old male Sprague–Dawley rats were given adaptive feeding for 2 weeks in a standard environment. All animal experiments were designed and executed in accordance with the Guidelines for Animal Health and Use of the National Institutes of Health and authorized by the Ethics Committee for Animal Experiments of Shanghai Jiao Tong University, Shanghai, China. After intraperitoneal injection of Zoletil (50 mg/kg, Virbac, France), the rats were anesthetized, followed by routine disinfection, hair removal, and incision. With the sagittal suture as the midline, circular bone drills with a 5 mm diameter were used to fabricate two symmetrical round defects on the parietal bone of rats (Figure 5A).

A total of 45 rats with bilateral skull defect area were randomly allocated into groups: negative control group, sham surgery group, COL group, EMC group, and HIMC membrane group. The membranes used to cover the defects were 9 mm × 9 mm square shape (2 mm beyond the defect edge), and in the negative control group, no membrane was placed in the defect area. For the sham surgery group, only the scalp was cut without the creation of a cranial defect. Cefazolin (10 mg/kg) was given to prevent infection for 3 days after the operation.

#### Micro-Computed Tomography Evaluation

For μ-CT, 20 rats with bilateral cranial defects were randomly allocated into four groups (*n* = 10 in each group). At 12 weeks post-surgery, the rats were sacrificed with excess Zoletil and the skull samples were fixed with 4% paraformaldehyde for 5 days. The samples were examined with a μ-CT system (Scanco Medical, Bassersdorf, Switzerland) under 70 kV voltage, 114 mA electric current, and 700 ms integration time. Considering the diameter and depth of the defect, we constructed a cylindrical profile to scan all materials and newly regenerated bone regions. For each sample, 150 consecutive cross-sections including the entire defect were collected. The image analysis software of the μ-CT 80 system was employed to calculate the ratio of bone volume to total tissue volume (BV/TV).

#### Histological Evaluation

##### Hematoxylin and Eosin Staining

After μ-CT scanning, these samples were collected and decalcified with 15% ethylene diamine tetraacetic acid (EDTA) for 3 weeks (*n* = 10 in each group), then gradually dehydrated, soaked in paraffin, and embedded. Next, the tissues were sliced into 5 μm-thick sections for HE staining. We used a stereoscopic microscope (Eclipse E600, Nikon, Tokyo, Japan) to obtain images. The proportion of new bone regenerated was calculated using Image-Pro Plus (7.0 version, Media Cybernetics, Rockville, MD).

##### Immunohistochemical Staining

For IHC staining, there were 25 rats divided into five groups (*n* = 10 in each group). The expression of osteogenic markers and macrophage polarization markers was detected after 2 weeks post-surgery. Sections were prepared as described above. Primary antibodies were added for incubation overnight including runt-related transcription factor 2 (Runx2, ab192256, dilution: 1:200, Abcam, Cambridge, MA, United States) and osterix (osx, ab22552, dilution: 1:200, Abcam, United States) as well as the macrophage polarization markers, pan marker CD68 (ab125212, dilution: 1:200, Abcam, United States), M1 marker iNOS (ab15323, dilution:1:100, Abcam, United States), and M2 marker CD206 (ab64693, dilution: 1:200, Abcam, United States). The sections were then incubated with secondary antibody for 1 h and stained with DAB. Finally, the nuclei were stained with hematoxylin. The stained sections were visualized and photographed under an optical microscope (Leica DMI 6000B Microsystems, Wetzlar, Germany). Four non-overlapping fields were randomly selected under 40x microscope for each section, and the number of nuclear-stained cells in the fields was considered positive staining cells.

### Statistical Analysis

All quantitative values are expressed as mean ± standard deviation (x ± SD). GraphPad Prism Software (Version 7.0, Inc., La Jolla, CA, United States) and SPSS 23.0 statistics software (IBM Corp, Armonk, NY, United States) were employed. Data analyses were performed with one-way analysis of variance (ANOVA) and Tukey’s multiple-comparisons test. The distribution normality of all datasets was evaluated by Shapiro-Wilk test. *p* values <0.05 (two-sides) were considered statistically significant.

## Results

### Comparison of the Physicochemical Properties of the HIMC, COL, and EMC Membranes

**FIGURE 1:**
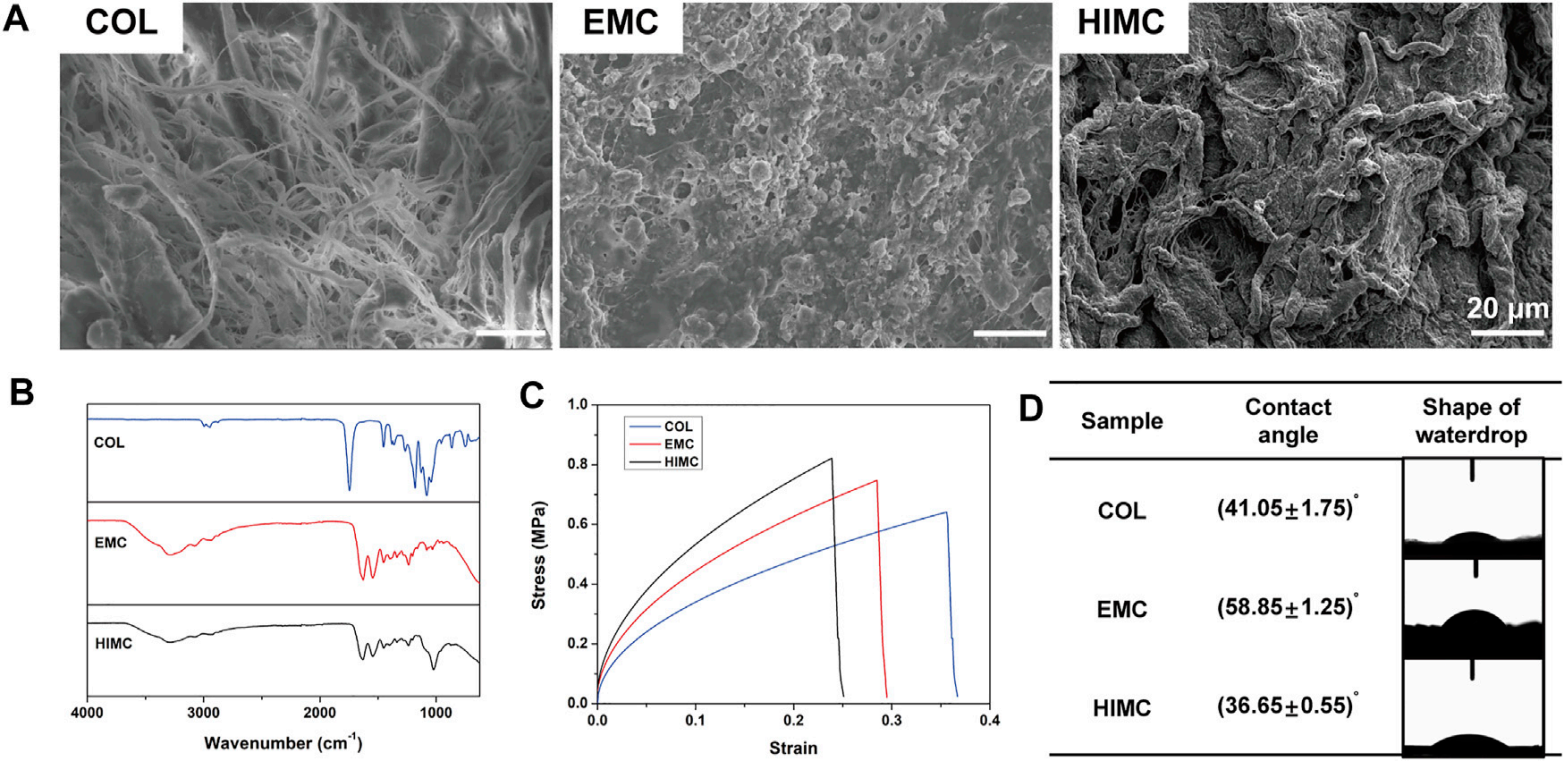
Characterization of the different membranes. **(A)** Representative surface topography images visualized by SEM. **(B)** FTIR spectra. **(C)** Stress–strain curves. **(D)** Contact angle and representative waterdrop images. Scale bar, 20 μm.

The microstructure of each material was observed by SEM. The HIMC membrane was structurally ordered and exhibited nanomorphology similar to the natural bone surface with a large surface roughness. The COL membrane exhibited a relatively smooth structure with no obvious protrusions. A coarse texture with nHA clusters stacked randomly around the fibers was observed on an EMC membrane (Figure 1A).

The functional chemical groups of the materials were detected by FTIR (Figure 1B). The spectra for all materials included peaks for amide I band (C=O bond, 1580–1720 cm^−1^) and amide II band (N=H bond, 1540 cm^−1^), which are the representative peaks of collagen. The presence of a phosphate vibration zone at 900–1200 cm^−1^ in the spectra for HIMC membranes represented mineralized particles.

The stress–strain curves obtained from the tensile testing showed that HIMC, EMC, and COL membranes exhibited similar deformation patterns (Figure 1C). The maximum tensile strength of HIMC was greater than that of the other membranes, and that of EMC was greater than that of COL.

Water contact angle measurements demonstrated the hydrophilicity of the three membrane types (Figure 1D). The contact angle appeared to be smallest on the HIMC membrane and largest on the EMC, indicating that the HIMC membrane was the most hydrophilic material, followed by COL and EMC. The difference in the contact angles was significant between the HIMC and EMC membranes and between the COL and EMC membranes, but not between the HIMC and COL membranes.

### Comparison of BMSCs Proliferation and Adhesion on HIMC, COL, and EMC Membranes

**FIGURE 2:**
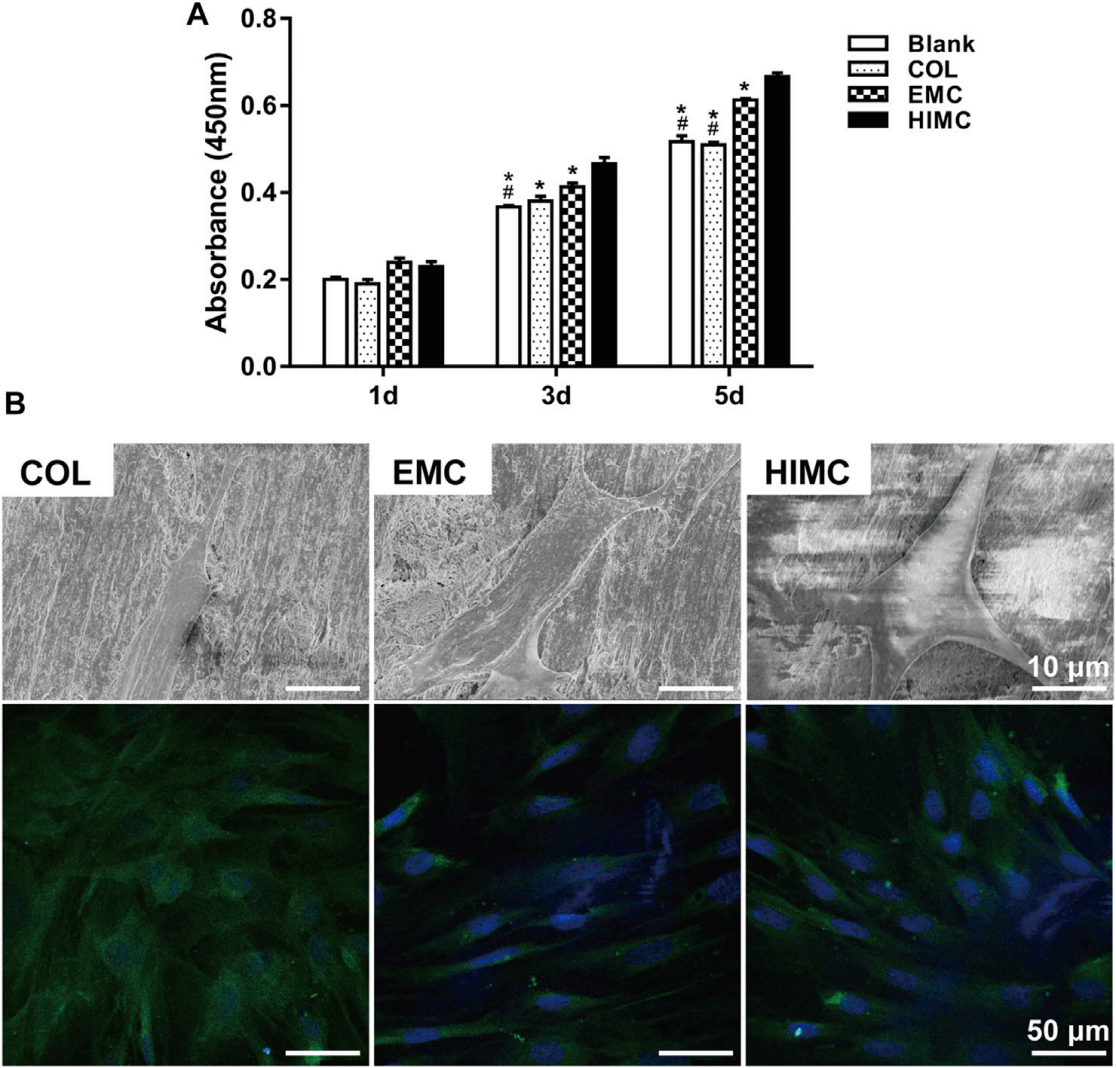
Growth and morphology of BMSCs cultured on the membranes. **(A)** CCK-8 assay results after 1, 3, and 5 days in culture. **(B)** Representative images of BMSC morphology after culture for 1 day, as visualized by SEM and LSCM. For LSCM images, immunofluorescence staining was performed for cytoskeletal proteins (green) and nuclei (blue). Data are expressed as mean ± SD. Scale bar, 10 μm in SEM and 50 μm in LSCM. ^*^ *p* < 0.05 vs. HIMC membrane; ^#^ *p* < 0.05 vs. EMC membrane.

The proliferation of BMSCs seeded on the HIMC, COL, and EMC membranes was compared using the CCK-8 assay (Figure 2A). On the first day of culture, no significant difference in absorbance at 450 nm was observed between the groups. Cell growth was not inhibited on all materials over time. On Day 3 and Day 5, significantly higher absorbance value was detected for the HIMC membrane group compared to the COL and EMC groups, showing that BMSCs seeding on HIMC membrane proliferated better than the others.

The morphology of BMSCs seeded on different membranes was visualized by SEM (Figure 2B). The BMSCs adhered to the HIMC membrane surface showed a polygonal shape with many large pseudopods extending outward. The BMSCs on the COL surface exhibited a thin spindle shape and lacked obvious pseudopod protrusion. Even fewer pseudopods that appeared small and thin presented on the BMSCs seeding on the EMC membrane.

The cytoskeletal organization within BMSCs seeded on different materials was observed by LSCM (Figure 2B). On COL membrane, BMSCs showed disordered cell fibers, with the cytoskeletal proteins oriented in different directions. On the EMC membrane, BMSCs had a fusiform shape, with elongated intracellular fibers, thin actin fibrils, and few branching points. In BMSCs on the HIMC membrane surface, the collagen fibers were tightly and thickly arranged, as seen in highly bifurcated osteoblasts, with a fine filamentous base and thick stress fiber formation.

### Comparison of BMSCs Osteogenic Differentiation on HIMC, COL, and EMC Membranes

**FIGURE 3:**
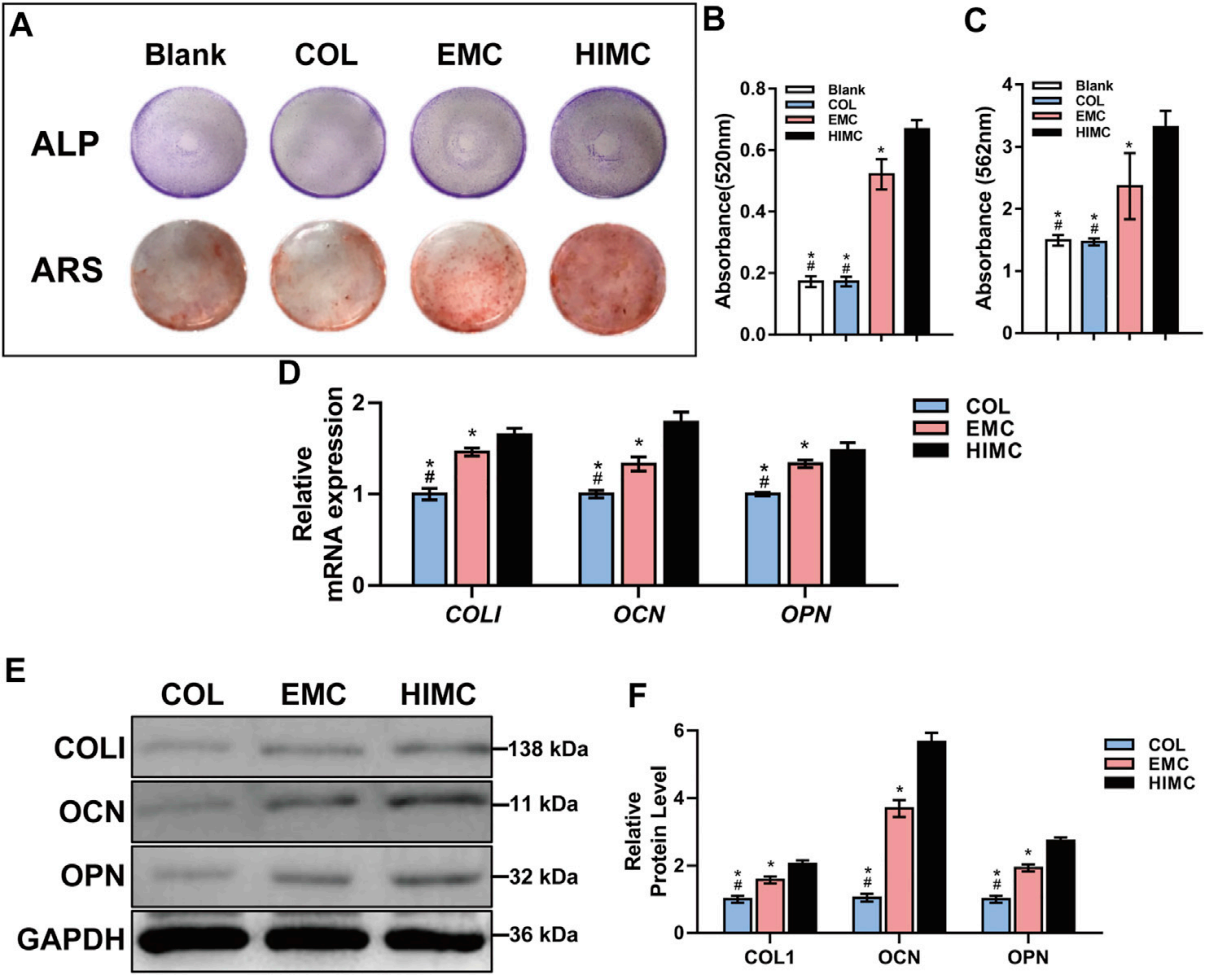
Osteogenic differentiation capacity of BMSCs cultured on different membranes. **(A)** ALP staining and ARS. **(B)** Semi-quantitative results of ALP activity assay for absorbance at 520 nm. **(C)** Absorbance at 562 nm for ARS. **(D)** mRNA expression levels of *COLI*, *OCN,* and *OPN* as measured by qRT-PCR. **(E)** Protein expression levels of COLI, OCN, OPN, and GAPDH as quantified by Western blot. **(F)** Gray-scale analysis of Western blots. Data are expressed as mean ± S.D. ^*^ *p* < 0.05 vs. HIMC membrane; ^#^ *p* < 0.05 vs. EMC membrane.

The osteogenic differentiation outcomes of BMSCs on different membranes were evaluated. BMSCs were cultured on different membranes for 14 and 21 days, and ALP and ARS were evaluated. For ALP activity assay, blue staining was more obviously observed on the HIMC membrane compared with COL and EMC membranes on Day 14 (Figure 3A). After 21 days in culture, ARS for calcium nodules presented densely red nodules among BMSCs on HIMC membranes, with fewer nodules on the EMC and COL membranes (Figure 3A). Semi-quantitative analysis of the results indicated the observed differences were statistically significant, the HIMC membrane distinctly enhanced the osteogenic capacity of BMSCs in terms of ALP and ARS (Figures 3B,C).

The mRNA and protein expression levels of osteogenic markers were measured in BMSCs cultured on different membranes for 3 days using qRT-PCR and Western blot analyses. BMSCs grown on HIMC membrane exhibited higher mRNA expression levels of the *COLI*, *OCN*, and *OPN* compared with BMSCs cultured on COL or EMC membranes (Figure 3D). ALP and COLI are early markers of osteogenesis, whereas OCN and OPN are expressed later in the osteogenic differentiation process. The results for protein expression obtained from Western blot were consistent with the qRT-PCR results (Figures 3E,F). Protein expression levels in the early (COLI) and late (OCN and OPN) osteogenic process were enhanced in BMSCs seeded on HIMC membranes compared with those in BMSCs seeded on COL and EMC membranes.

### Comparison of Macrophage Polarization and Consequent BMSCs Migration on HIMC, COL, and EMC Membranes

**FIGURE 4:**
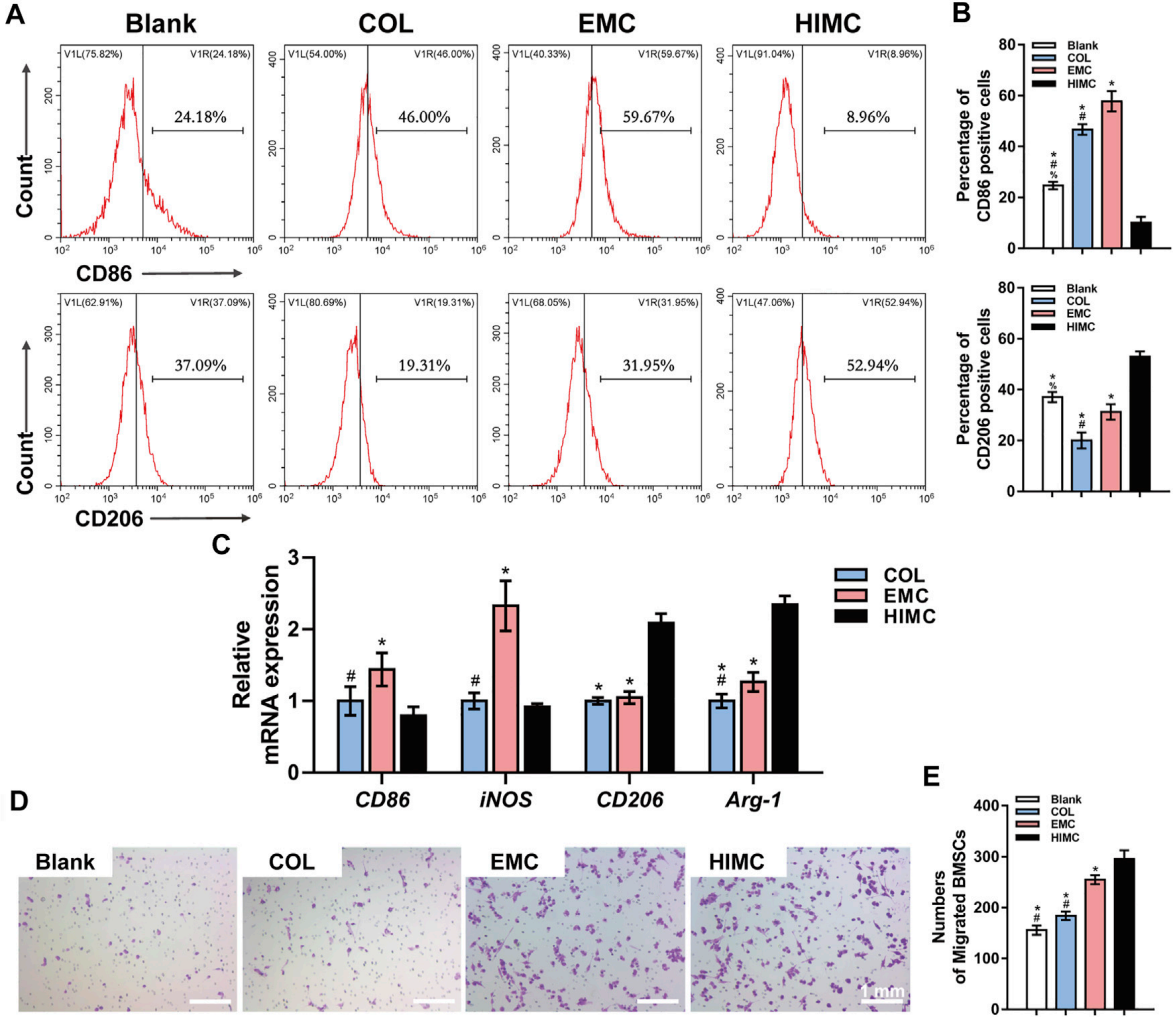
Macrophage polarization and BMSC migration in response to different membranes. **(A)** Representative peak plots of CD86^+^ (M1 polarization) and CD206+ (M2 polarization) macrophage ratios examined by flow cytometry. **(B)** Percentages of CD86^+^ and CD206 + cells. **(C)** mRNA expression levels of M1 polarization genes (*CD86, iNOS*) and M2 (*CD206, Arg-1*) in macrophages as measured by qRT-PCR. **(D)** Crystal violet staining of Transwell inserts for detection of BMSC migration induced by macrophages. **(E)** Quantification of BMSC migration. Data are expressed as mean ± SD. Scale bar, 1 mm. ^*^ *p* < 0.05 vs. HIMC membrane; ^#^ *p* < 0.05 vs. EMC membrane; ^%^ *p* < 0.05 vs. COL membrane.

To investigate the immunoregulatory characteristics of HIMC membrane, we exposed RAW 264.7 cells to HIMC, COL, and EMC membrane. Flow cytometric analysis was performed to identify M1 and M2 macrophage types after culture on different materials, based on the proportion of CD86^+^ and CD206 + cells (Figures 4A,B). The percentages of CD206 + macrophages among different groups were 52.94% with HIMC membrane, 31.95% with EMC, and 19.31% with COL, and from these results, the percentage of M2 macrophages in the HIMC membranes group was remarkably higher. The percentages of CD86^+^ macrophages were 8.96% with HIMC membrane, 59.67% with EMC, and 46% with COL, indicating that fewer macrophages in the HIMC group showed M1 polarization. Therefore, with the presence of more M2 macrophages and fewer M1 macrophages, the HIMC membrane was associated with the largest M2/M1 macrophage ratio among the three tested materials.

The relative mRNA expression levels of *CD86*, *iNOS* (M1 polarization markers), *CD206,* and *Arg-1* (M2 polarization markers) in RAW 264.7 cells exposed to different membranes were detected by qRT-PCR (Figure 4C). Compared with the levels in the other two groups, the expression levels of *CD86* and *iNOS* in the HIMC membrane group were greatly reduced, and *CD206* and *Arg-1* were notably increased. These results were similar to those from flow cytometric analysis, and macrophages on the HIMC membrane showed more M2 polarization.

Next, we studied the effects of different materials on interaction between BMSCs and macrophages (Figure 4D). First, macrophages were stimulated by conditioned medium containing leached materials from the different membranes, and then macrophages and BMSCs were co-cultured in Transwell chambers for 24 h. In this assay, the number of BMSCs that migrated from the upper to the lower layer was statistically greater in the HIMC membrane group than the COL membrane group (Figure 4E), although no difference was detected between the EMC and HIMC groups.

### Comparison of Bone Regeneration in Critical-Sized Skull Defect Model Covered With HIMC, COL, or EMC Membranes

**FIGURE 5:**
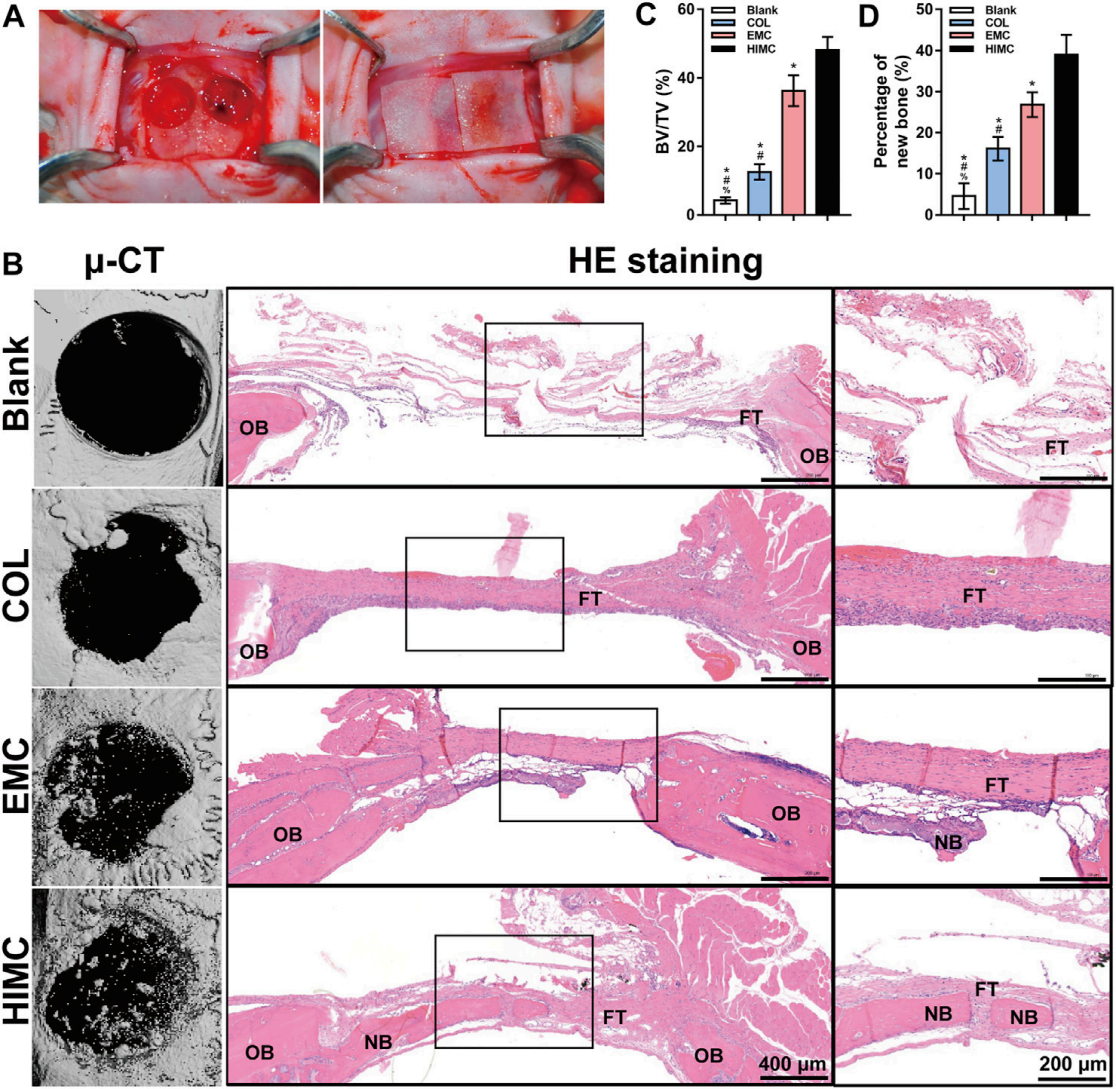
Bone regeneration in rat critical-sized skull defect models covered with the different membranes. **(A)** Rat critical-sized cranial defect models. **(B)** Representative μ-CT images, HE staining images, and ×40 magnification images of the regions outlined by black rectangles at 12 weeks post-surgery. **(C)** Quantitative analysis of new BV relative to the TV on the basis of μ-CT. **(D)** Semi-quantitative analysis of the percentages of new bone in the defect according to HE staining. Data are expressed as mean ± SD. Scale bar, 400 μm (20×) and 200 μm (40×). ^*^ *p* < 0.05 vs. HIMC membrane; ^#^ *p* < 0.05 vs. EMC membrane; ^%^ *p* < 0.05 vs. COL membrane.

To assess the osteogenic ability of HIMC membrane *in vivo* GBR model, we prepared critical-sized skull defects in rats and then covered the defects with different membranes (Figure 5A). μ-CT scanning was performed at 12 weeks post-surgery (Figure 5B). In the HIMC membranes group, nearly mature bone structure filled most of the defect area, and the density of the newly formed bone was analogous to that of the surrounding bone tissue. Bits of new bone were regenerated at the defect edges of the COL membrane group. In the EMC group, the defect area was reduced, and a network with a low-density shadow had formed in some areas. By comparison, in the negative control group which defects were not covered with membranes, the bone defect surface appeared round and smooth, with little new bone tissue presented at the defect edge and no obvious mineralized structures were observed. Quantitative evaluation of the μ-CT images discovered that the ratio of BV/TV in the HIMC membrane group (0.4818 ± 0.0574) was statistically higher than those in the EMC (0.3627 ± 0.0436; *p* < 0.05) and the COL group (0.1252 ± 0.0196; *p* < 0.05; Figure 5C).

HE staining of the harvested skull defect area produced results consistent with the those from μ-CT imaging (Figure 5B). The HIMC membrane group showed newly formed mineralized bone with a higher density of bone trabecula and more mature structures as well as a significantly reduced defect area, compared with the other groups. In the stained section from the HIMC membrane group, abundant osteocytes and new bone distributed from the edge of the defect to the center, and a typical bone marrow cavity structure could be seen. In contrast, staining of the new bone in the COL group and the EMC group showed a lower trabecular density, with new bone found only at the defect edge. In the negative control group, the defect area was predominantly fibrous tissue with no evidence of new bone formation. The semi-quantitative analysis results of the percentage of new bone based on HE staining images are presented in Figure 5D. The new bone percentage in the HIMC membrane group (39 ± 4.43%) was remarkably higher than those in the EMC membrane (26.83 ± 2.74%; *p* < 0.05) and the COL membrane group (16.12 ± 2.6%; *p* < 0.05).

**FIGURE 6:**
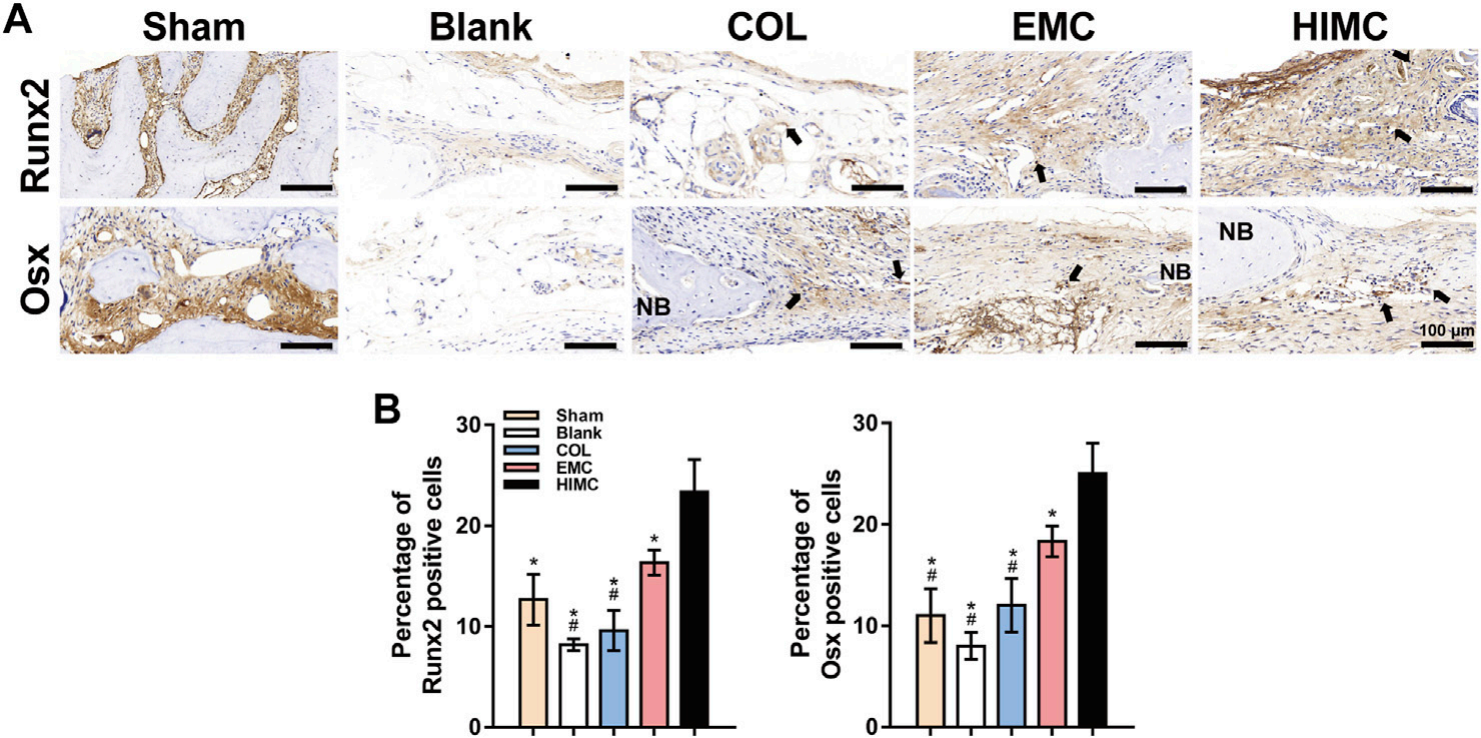
Representative images of IHC staining for osteogenesis markers in the defect region of different groups at 2 weeks post-surgery. Positively stained cells are indicated by arrows. **(A)** Runx2 and Osx expression in defect tissues. **(B)** Semi-quantification of the positive cells. Data are expressed as mean ± SD. Scale bar, 100 μm. ^*^ *p* < 0.05 vs. HIMC membrane; ^#^ *p* < 0.05 vs. EMC membrane; ^%^ *p* < 0.05 vs. COL membrane.

IHC staining was performed to assess the performance of two transcription factors related to osteogenesis, Runx2 and Osx, in the bone defect area (Figure 6A). HIMC membrane samples contained more positive cells that highly expressed Runx2 and Osx, whereas a small number of positively stained cells were found in the samples from the COL and EMC groups. No significant positive staining was observed in either the negative control or the sham group. Semi-quantitative analysis of the IHC staining images presented statistical differences between the groups (Figure 6B).

### Comparison of Macrophage Polarization in Critical-Sized Skull Defect Model

**FIGURE 7:**
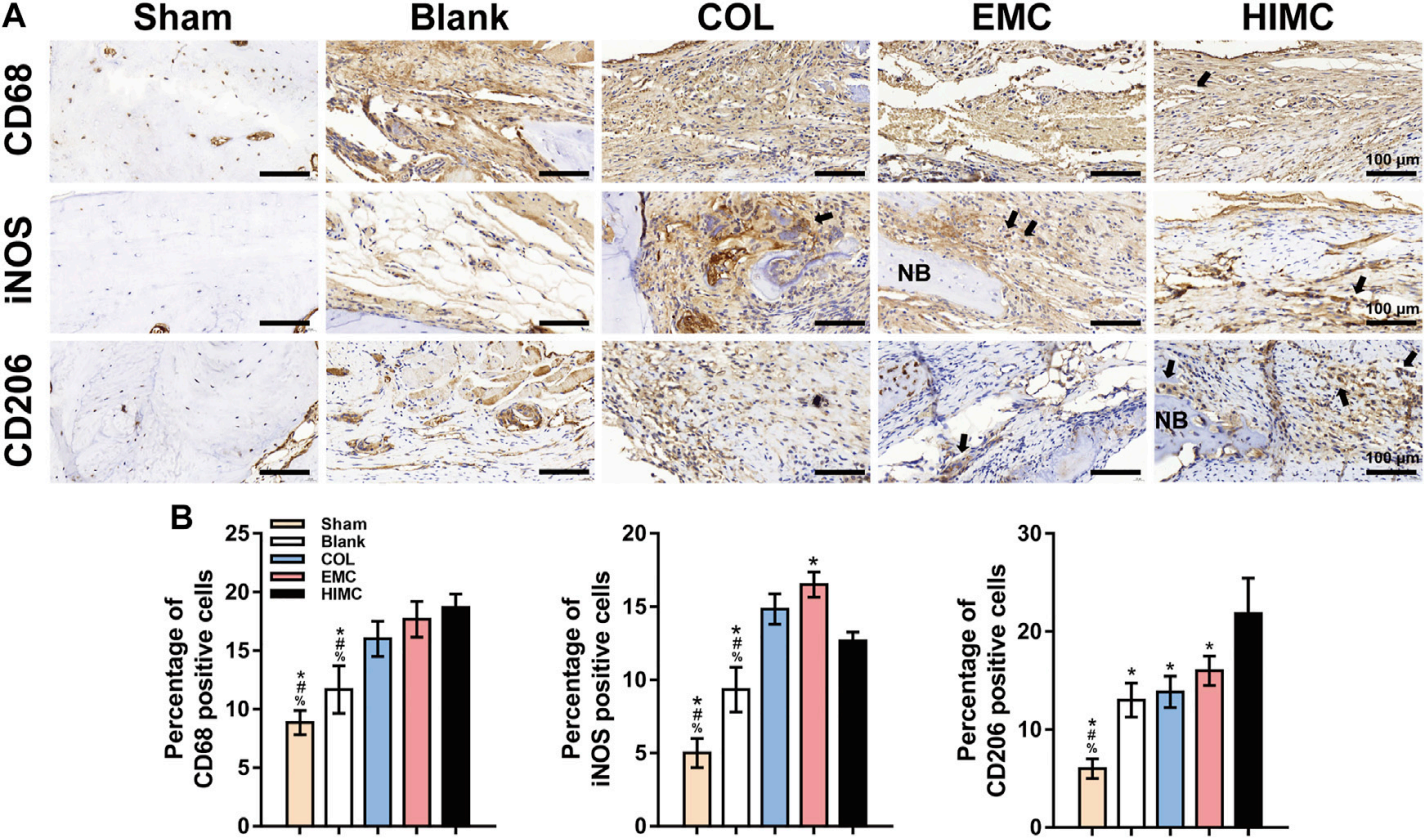
IHC staining for macrophage polarization markers in the defect region of different groups at 2 weeks post-surgery. Positively stained cells are indicated by arrows. **(A)** Pan marker CD68, M1 marker iNOS, and M2 marker CD206 expression in defect tissues. **(B)** Semi-quantification of the positive cells. Data are expressed as mean ± SD. Scale bar, 100 μm. ^*^ *p* < 0.05 vs. HIMC membrane; ^#^ *p* < 0.05 vs. EMC membrane; ^%^ *p* < 0.05 vs. COL membrane.

To evaluate the osteoimmunomodulatory properties of the HIMC membrane in the bone defect area, we examined the polarization status of macrophages by IHC staining (Figure 7A). CD68 is a pan marker for *in situ* macrophages. CD68^+^ cells were observed in samples from all groups except the sham group, indicating that macrophages played a role in the response to the materials in the bone defect. Macrophage phenotype was examined by IHC staining for M1 (iNOS) and M2 (CD206) markers. More iNOS + cells were stained in the EMC group, in a pattern consistent with CD68 ^+^ cells, revealing that most of the macrophages in the defects covered with EMC membrane possessed the M1 phenotype. Fewer iNOS + cells were observed in the samples from the COL group and the HIMC membrane groups. On the other hand, we observed the largest number of CD206 + cells on the HIMC membrane group, further confirming the dominant effect of M2 macrophages in the defects covered with the HIMC membrane. The results of semi-quantitative analysis of the percentage of positive cells on IHC-stained images are presented in Figure 7B.

## Discussion

Biomimetic mineralized materials have been widely applied in studies of tissue and organ regeneration, and the prepared membranes have shown some ability to promote GBR (Fillingham and Jacobs, 2016; Yu et al., 2020). However, previous EMC material was commonly characterized by irregular HA deposition. Compared with EMC, the HIMC membrane was shown to more successfully simulate the nanostructure of natural bone through intrafibrillar mineralization and have a better ability to induce osteogenesis (Liu et al., 2016; Wang et al., 2019). According to the previous research, intrafibrillar mineralization is the main structural source of the biomechanical properties of bone and affects the biological activity of relevant cell types (Balooch et al., 2008). In the present study, we fabricated HIMC membranes with a composite structure consisting of collagen and HA to guide bone regeneration. Compared with EMC and COL, the HIMC membrane exhibited a clear structure and coarse surface on SEM, with regular interspersion of rigid nHA and flexible collagen, providing superior strength similar to that of natural bone. Tensile testing results also showed that the HIMC membrane exhibited less strain under the same stress compared with EMC and COL. On FTIR spectra reflecting the chemical composition and mineral spatial distribution of the materials, the typical peaks for collagen and phosphate were observed for the HIMC membrane. Water contact angle measurements suggested that the HIMC membrane was more hydrophilic and, thus, was more conducive to interaction between the membrane material and host fluids and cells upon implantation.

To evaluate the potential value of the HIMC membrane for promoting GBR, we first observed the behaviors of BMSCs seeded on different materials *in vitro*. The composition of biomaterials is known to affect cell morphology and adhesion (Ayala et al., 2011; Perez and Ginebra, 2013). BMSCs cultured on the HIMC membrane showed greater proliferation potential and better adhesion and morphology compared with those cultured on EMC or COL. SEM further showed that BMSCs extended large pseudopods on the HIMC membrane surface, whereas little expansion was observed on the other materials. A highly branched actin cytoskeleton and the formation of stress fibers are thought to be highly associated with the differentiation of stem cells along the osteogenic lineage, moreover promote the intracellular signal transduction (Mathieu and Loboa, 2012; Müller et al., 2013; Fu et al., 2016). Our immunofluorescence staining results confirmed that cell fibers within the BMSCs adhered to the HIMC membrane were thick and densely arranged, which may make for BMSCs osteogenic differentiation.

Next, we verified the osteogenic induction capacity of the HIMC membrane through *in vitro* and *in vivo* experiments. The outcomes of our research consistently proved that interaction with HIMC membrane up-regulated the osteogenesis-related genes and proteins in BMSCs during both the early and the late stages of osteogenesis. ARS also showed more calcium deposition and mineralized nodules among BMSCs adhered to the HIMC membrane *in vitro*. In the rat model of critical-sized skull defect, μ-CT and histological staining showed that by 12 weeks after application, the HIMC membrane within the defect had been essentially replaced by new bone tissue, which was not observed in defects filled with EMC or COL. Together the results of this study indicated that the HIMC membrane significantly promoted more bone regeneration and supported the GBR process both *in vivo* and *in vitro*.

Bone defect repair is a dynamic physiological process, and prior to osteogenesis and angiogenesis, the early inflammatory response of immune cells to biomaterials is a major determinant of treatment outcome (Franz et al., 2011). After implantation of biomaterials within a bone defect, osteocytes and immune cells partake a mutual microenvironment (Vishwakarma et al., 2016). As the central regulator of cellular activity within the bone defect area, the immune microenvironment, especially the macrophages affect the efficacy of biomaterial therapy. Previous research has proven that the M1 and M2 phenotype macrophages participate in the early inflammatory response and later bone wound healing, respectively, and that the M2/M1 ratio reflects the response of the local immune microenvironment (Ma et al., 2015; Wood et al., 2019).

CD86 is a costimulatory receptor necessary for T cell activation. CD206, also known as mannose receptor C type 1 (MRC1), is a cell-surface protein abundantly present on macrophages. As for macrophages, CD86 is normally expressed on M1 subtype, while CD206 is expressed on M2 (Barros et al., 2013; Fuchs et al., 2016). Our flow cytometric analysis showed that the rates of CD86^+^ (M1) and CD206+ (M2) macrophages exposed to HIMC membrane were 8.96 and 52.94%, which corresponded to the highest percentage of M2 macrophages and the highest M2/M1 ratio. mRNA expression analysis also revealed lower M1 expression and higher M2 expression of cells seeded on the HIMC membrane, further indicating that the macrophages exhibited more M2 polarization on the HIMC membrane. Otherwise, CD163 has also been suggested as an M2 marker, but more recently was shown that CD163 is an M2 marker only in combination with the transcription factor cMaf, thus CD163 cannot be considered as an M2 polarization marker when used alone (Barros et al., 2013). CD68 is a protein found in the granules of macrophages, which is used to co-label cells positive for a certain marker after IHC or *in situ* hybridization to prove they are macrophages (Gordon et al., 2014). Our results from IHC staining of skull defect samples were consistent with those from flow cytometry and qRT-PCR analyses, the HIMC membrane induced more CD68 + CD206 + macrophages polarization. In summary, these data concluded that the HIMC membrane induced more M2 phenotype macrophages both *in vivo* and *in vitro*. Zhou et al. previously reported pro-inflammatory response and damaged lysosomes on macrophages in EMC. Large HA particles impair the normal structure of cells, which may explain why EMC induced more M1 polarization (Jin et al., 2019). Therefore, ordered nHA particles on the HIMC membrane may create a better anti-inflammatory environment.

During normal fracture healing, pro-inflammatory M1 macrophages gradually transform into anti-inflammatory M2 macrophages, which corresponds to the regression of inflammation and the initiation of the osteogenesis process (Schlundt et al., 2018; Zhang et al., 2019). The transformation from M1 to M2 macrophages also contributes to the recruitment of MSCs and the consequent osteogenesis differentiation (Gibon et al., 2016; Weitzmann and Ofotokun, 2016). We investigated the effects of immune microenvironments including macrophages and different materials on the migration of BMSCs via Transwell experiments. Results showed that the HIMC membrane induced migration of the highest number of BMSCs, indicating that the HIMC membrane immune microenvironment may be better capable of recruiting MSCs. Moreover, M2 macrophages on the HIMC membrane express crucial genes to promote BMSCs differentiation such as interleukin (IL)-4 (Jin et al., 2019; Mahon et al., 2020). On another hand, scaffolds loaded with IL-4 for the purpose of promoting M1 to M2 polarization showed promising outcomes in fracture repair models, further demonstrating the beneficial effect of M2 in bone regeneration (Schlundt et al., 2018). To sum up, these findings demonstrated that the HIMC membrane acted not only on MSCs directly, but also more importantly affected the process of osteogenesis by regulating macrophage polarization.

The osteoimmunomodulatory effects of biomaterials are also significantly affected by the physicochemical properties of the materials, like the surface morphology, porosity, and hardness (Chen et al., 2018; Sadowska et al., 2018; Li et al., 2020). The concept of “nano-bone immunoregulation” proposed by Xiao et al. emphasizes the adjustment of the chemistry and morphology of a nanostructure surface in order to influence the immune response in bone regeneration applications (Karageorgiou and Kaplan, 2005; Chen et al., 2017; Yu et al., 2018). The HIMC membrane prepared in the present study had a coarser surface and BMSCs showed better adhesion and extension on this membrane. These structural features were found to effectively promote the growth and osteogenic differentiation of BMSCs. In addition, the macrophages exhibit greater contact with the increasing roughness of biomaterials. Thus, cell adhesion, migration, proliferation, and differentiation can be directly influenced by precisely controlled changes in the tomography of the biomaterial surface. The underlying mechanism involves the effects of physicochemical interactions, kinetics, and thermodynamic exchanges between nanotopography and biological systems on macrophage morphology and the transfer of physicochemical signals from outside to inside a cell to activate a variety of biological reactions (Chen et al., 2017). Therefore, rationally designed nanomaterials offer a promising strategy for enhancing bone regeneration and osteoimmunomodulatory efficacy.

To date, many studies have explored the modification of mineralized collagen materials. One strategy involves adding inorganic components to achieve dopant-induced osteogenesis, and collagen has been loaded or coated with HA modified with metal ions, like magnesium (Yu et al., 2018), silver, gold (Kumar et al., 2019), and zinc (Tiffany et al., 2019). The presence of these metal ions with the biomaterial creates a micro-current effect, which synergistically affects osteogenesis and immune microenvironment (Cai et al., 2017). Overall, the research to date indicates that composite materials with functional modifications are the direction in future biomaterials development. Meanwhile, the next generation of biomaterials for GBR, the design paradigm should shift from physical structures to bioactive structures with osteoimmunomodulatory properties.

The present study has limitations to consider. The concrete data involving osteogenic effects and macrophages polarization of the HIMC membrane remains unclear. The molecular mechanism by which HIMC membrane promotes macrophage polarization is also needed to elucidate. Further investigation will be required in the future.

## Conclusion

Based on our findings, the HIMC membrane provided a favorable immune microenvironment for M2 macrophage polarization and osteogenic differentiation of BMSCs by mimicking the composition and nanostructure of natural bone. As a result, the HIMC membrane promotes bone regeneration and plays osteoimmunomodulatory effects, and hence represents a promising membrane material for GBR.

## Data Availability

The original contributions presented in the study are included in the article/Supplementary Material, further inquiries can be directed to the corresponding authors.
