# Supplementary figures and images for: Hierarchical Intrafibrillarly Mineralized Collagen Membrane Promotes Guided Bone Regeneration and Regulates M2 Macrophage Polarization
Source: Front Bioeng Biotechnol. 2022 Jan 26;9:781268. doi: 10.3389/fbioe.2021.781268 (PMC8826568; doi:10.3389/fbioe.2021.781268)

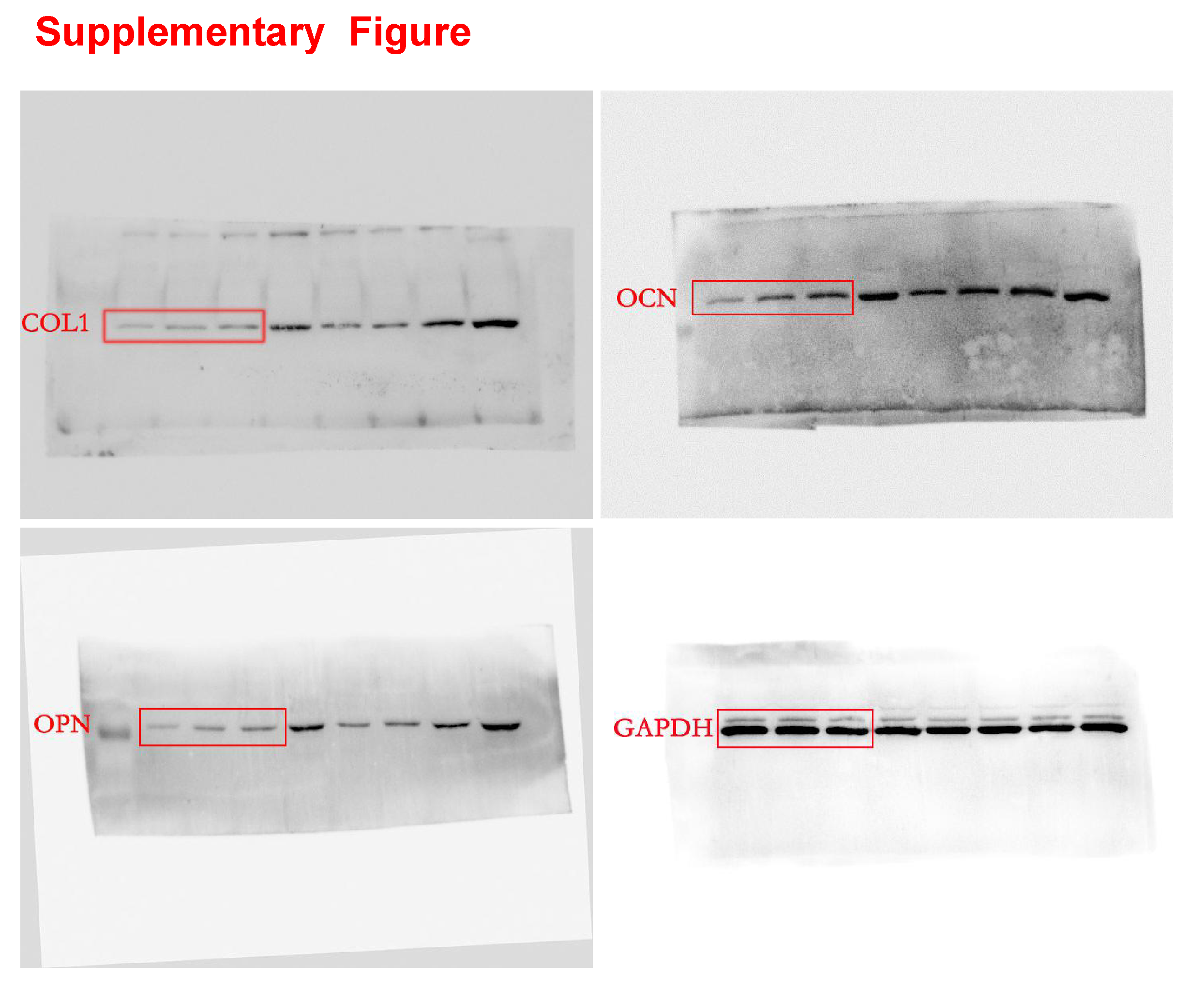

Supplement: Supplementary file 1 [file Image1.TIF]
